# Therapy of aseptic nonunions with parathyroid hormone

**DOI:** 10.1007/s00590-018-2269-9

**Published:** 2018-06-21

**Authors:** I. Kastirr, M. Reichardt, R. Andresen, S. Radmer, G. Schröder, T. Westphal, T. Mittlmeier, H. C. Schober

**Affiliations:** 1 Institute of Diagnostic and Interventional Radiology/Neuroradiology, Westkuestenklinikum Heide, Academic Teaching Hospital of the Universities of Kiel, Lübeck and Hamburg, Esmarchstr. 50, 25746 Heide, Germany; 2 Department of Internal Medicine I, Municipal Hospital Suedstadt Rostock, Academic Teaching Hospital of the University of Rostock, Rostock, Germany; 3 Center of Orthopaedics, Berlin, Germany; 4 Clinic of Trauma Surgery, Orthopaedics and Hand Surgery, Municipal Hospital Suedstadt Rostock, Academic Teaching Hospital of the University of Rostock, Rostock, Germany; 5 Clinic of Trauma, Hand- and Reconstructive Surgery, Rostock University, Medical Center, Rostock, Germany

**Keywords:** Aseptic nonunions, Attempted treatment, Fracture healing, Parathyroid hormone

## Abstract

The absence of osseous consolidation of a fracture for 9 or more months with no potential to heal is defined as nonunion. Both for the patient and from a socioeconomic point of view, nonunions represent a major problem. Hypertrophic, vital nonunions are distinguished from atrophic avital ones. Risk factors for a delayed fracture healing are insufficient immobilisation, poor adaptation of the fracture surfaces or residual instability, interposition of soft tissue within the fracture gap, as well as circulation disturbances and infections. The incidence of nonunions after fractures of the long bones lies between 2.6 and 16% depending on the surgical technique used. In human and animal studies, a positive effect of parathyroid hormone (PTH) on fracture healing has been shown. PTH has a direct stimulatory effect on osteoblasts and osteoclasts. In addition, it appears to influence the effect of osseous growth factors. In this prospective study, 32 patients with nonunions were treated with teriparatide to investigate the effects of PTH on fracture healing. Definitive healing of the nonunions following PTH treatment could be observed in 95% of the cases.

## Introduction

Most definitions found in the literature regarding nonunions assume that this diagnosis should be made when no healing has occurred within 9 months of a fracture incident with no visible signs of progressing consolidation in conventional radiographs, as distinct from the broader notion of delayed unions, defining the lack of restoration of osseous continuity of a fractured bone after a normal period of time typical for the fractured bone.

The development of nonunions is not rare, occurring at a rate of 2.6–16% in fractures of the long bones both after conservative therapy and surgical treatment [1]. For the patient, this means limited weight-bearing capacity and loss of muscle mass and power, persistent pain and thus a loss of quality of life, reduced productivity and potentially a loss of income. On the other side, the healthcare system is burdened with costs for further operations, medication, hospitalisation and absence from work [2].

The healing of a bone after a fracture requires a well-regulated interplay of biochemical and biomechanical processes. In each phase of the fracture repair, disturbances can occur that impair or interrupt bone healing. Large-scale necrosis, a lack of vascularisation or poor circulation due to diabetes mellitus or peripheral arterial occlusive disease, but also infections can already impair callus formation in the inflammatory phase. If there is a lack of fracture healing accompanied by insufficient callus formation, one refers to an oligotrophic or atrophic nonunion [2–4].

Inadequate immobilisation of the fracture can impede healing. Excessive movement of the fracture ends can promote the formation of fibrous tissue in the fracture gap and prevent ossification [5]. As a reaction to instability, an excessive formation of callus tissue around the fracture gap is often seen, but this does not correspond to better stability. In these cases, one refers to hypertrophic nonunion which makes adequate stabilization after debridement necessary [3, 5].

Other biochemical factors can have a negative effect on fracture healing, e.g., a vitamin D deficiency can prevent calcification of the bone matrix formed. Medications such as corticosteroids, nonsteroidal anti-inflammatory drugs and cytostatics can have an inhibitory effect on fracture healing. Negative effects have also been described for osteoporosis, malnutrition, anaemia or chronic hypoxia [3].

Ultimately, increased age of the patient is also a negative influencing factor, since the capacity of the osteoprogenitor cells to divide and differentiate decreases with advancing age, and the angiogenic capacity in the bone declines [6].

Implantation of autologous bone graft has been the gold standard in treating nonunions for many years. To improve bone healing but reduce the risk of complications at the explantation site, other local procedures such as the transplantation of allogeneic bone or bone substitute materials like decalcified bone matrix have been used. More recently, the injection of biological substances like bone morphogenic proteins (BMPs) into the fracture gap intraoperatively have shown great potential but also have raised questions about complications and cost. Most recently, cell-based therapies are being investigated. They include harvesting and injection of precursor like mesenchymal stem cells [7]. These procedures require further surgical interventions that would go along with further risk of complications, immobilisation, etc.

The experimental use of osteoporosis medications such as bisphosphonates in animal studies has shown that these can have a positive effect on callus size and durability [8, 9]. However, these substances inhibit bone resorption and may thus have an inhibitory effect in the phase of remodelling, in fact, it has been shown that they inhibit callus remodelling in animal studies [2, 6, 7, 9, 10].

For more than 10 years, parathyroid hormone has been used for the treatment of osteoporosis and the reduction in fracture risk. In two controlled, double-blind, randomised studies, an accelerating effect of PTH on the healing of pelvic and distal radial fractures has been observed in postmenopausal women [11, 12]. In various different animal experimental studies of different fracture models, it was shown that the administration of PTH accelerates fracture healing and increases the callus volume and mineral salt content in the fracture area, while simultaneously increasing the resistance to traction [6, 10].

Parathyroid hormone intervenes at different points of bone metabolism. On the one hand, by stimulating vitamin D synthesis in the kidneys, it leads to an increased uptake of calcium from the intestines, which is required for mineralisation of the bone matrix. On the other hand, it has been shown that PTH can stimulate the differentiation of osteoblasts and chondroblasts by stimulating the expression of BMPs [13]. Whereas the physiological effect mainly comprises an activation of the osteoclasts for bone resorption, intermittently administered PTH can lead to a shift in balance of bone resorption and bone formation via a direct activation of osteoblasts in the direction of osteogenesis [2, 6, 10, 13].

PTH thus plays a central role in the coupling of osteogenesis and osteolysis and is an important regulator in the process of remodelling. These properties suggest that PTH might be suitable for application in the treatment of nonunions. A possible positive effect of PTH on the healing of nonunions has already been observed in a number of case reports [2, 6, 14–19].

In the following, we report about the off-label use of teriparatide as a trial to treat nonunions. Both hypertrophic and hypotrophic nonunions were included. Cases where manifest mechanical instability was identified as the reason for hypertrophic nonunion were excluded.

### Patients and methods

Thirty-two patients, 15 male and 17 female, within the age of 22–83 (55.1 ± 15.9) years who had developed a nonunion were included in the study (Table 1). Inclusion criteria were absence of infection validated by normal body temperature, blood leucocyte count and C-reactive protein (CRP) as well as absence of vitamin D deficiency with serum levels of > 30 ng/ml and disturbances in the calcium phosphate metabolism. The interval from the last surgical intervention had to be 3 months at minimum. The fracture locations were at the tibial pilon (*n* = 16), the femur (*n* = 8), the ankle joint (*n* = 2), the distal humerus (*n* = 1), the olecranon (*n* = 1), the distal radius (*n* = 1), the distal tibia (*n* = 2) and at the metatarsal bone level (*n* = 1). The patients underwent an average of two operations (0–14) before treatment was started. Twelve patients suffered from hypertension, seven from obesity (BMI > 29.9 kg/m^2^), diabetes mellitus was known in three patients (type I *n* = 2, type II *n* = 1). Two patients had chronic kidney disease (stage I), three patients each had hyperthyreosis and hypothyreosis, respectively. Seven patients had autoimmune diseases (rheumatoid arthritis, psoriasis or other types of collagenosis), and two patients of them were under permanent treatment with glucocorticoids.

**Table 1:** Distribution of age, sex and fracture sites (mean ± SD), as well as number of operations (median) and chronic diseases among patients (total number)

Age (years)	22–83 (54 ± 15.9)
Sex (female/male)	17/15
Fracture sites	Pilon tibial (*n* = 16), malleolus (*n* = 2), distale crurale (*n* = 2), femoral (*n* = 8), metatarsale (*n* = 1), distal humerus (*n* = 1), olecranon (*n* = 1), distal radius (*n* = 1)
Operations	2 (0–14)
Chronic diseases (number of patients, including double counts in multimorbid patients)	Hypertension (12), obesity (7), diabetes mellitus type I (2), type II (1), chronic kidney disease stadium I (2), hyperthyreosis (3), hypothyreosis (3), autoimmune diseases (7)

The off-label character of the PTH treatment as well as possible side effects was explained to the patients before initialization of treatment with PTH, while all of them consented with this particular treatment option eventually substituting a further surgical intervention. Informed consent was obtained from each subject, and all procedures were performed in accordance with the Declaration of Helsinki. Treatment costs were carried by the patients or by the treating clinic. Patients were treated for 4–10 (7.3 ± 1.5) weeks with 20 µg of teriparatide, the 1–34 fragment of recombinant human parathyroid hormone (Forsteo®, Lilly Deutschland GmbH, 61352 Bad Homburg) per day. Therapy was continued until declining pain under weight-bearing conditions and manifestations of bone bridges in the fracture gaps with or without increasing callus volume in standard radiographies in two planes occurred.

Clinical examination including pain assessment under full weight-bearing or loading of the corresponding extremity, blood analysis (concentration of calcium, phosphate, creatinine, CRP, blood cell count) and conventional radiographs in two planes were carried out every 4 weeks.

Endpoint of the study was the pain-free weight-bearing capacity of the corresponding extremity and the consolidation of the nonunion proven by bone bridges in at least 3/4th of the previous fracture gap and the surrounding cortical bone seen in conventional radiographs.

### Statistics

Descriptive statistics included the calculation of the mean values ± standard deviations apart from the numbers of surgical interventions where we preferred the calculation of the median value, which better reflects the clinical status prior to the initialization of PTH treatment.

## Results

**Fig. 1:**
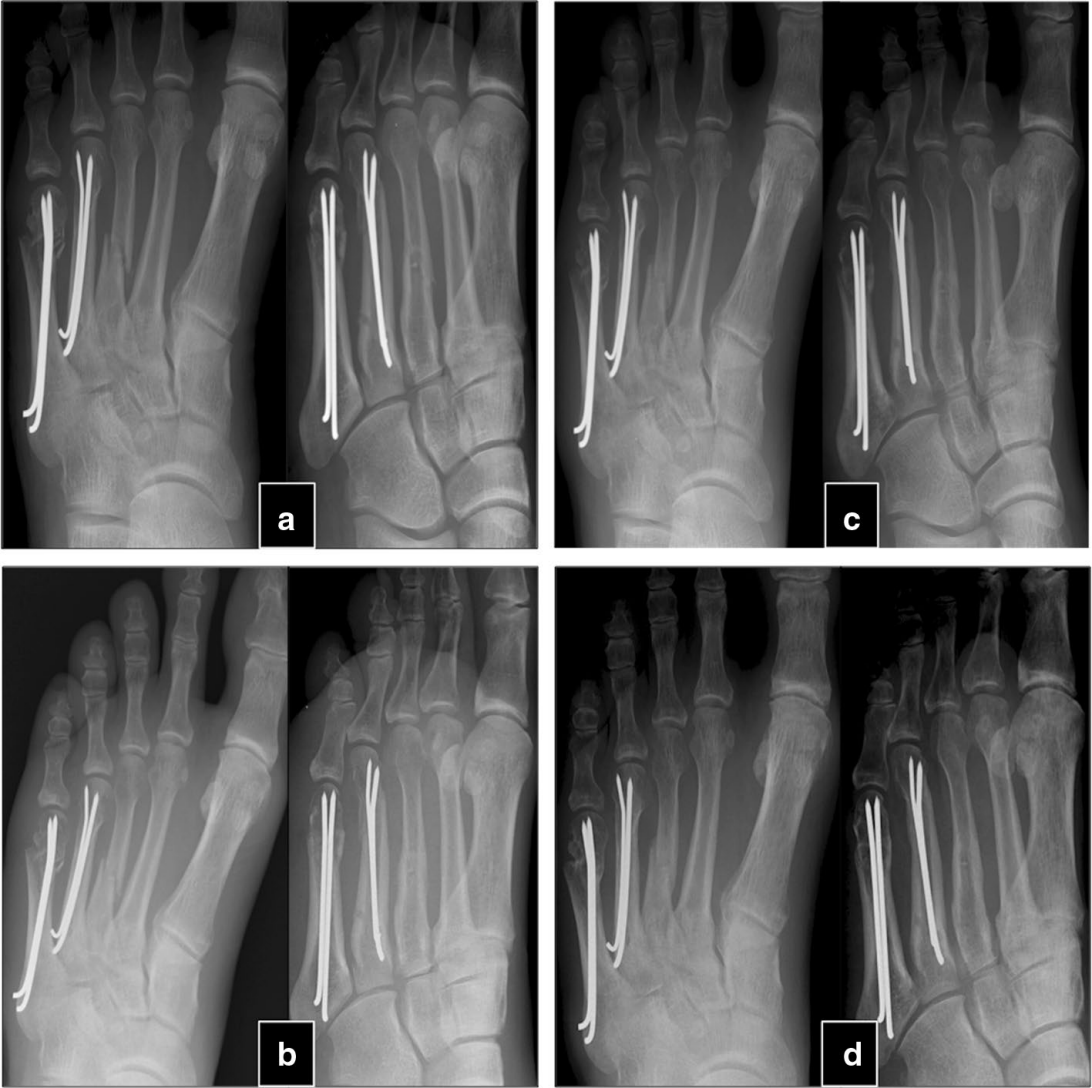
Fifty-eight-year-old male smoker with treated arterial hypertension with foot injury in a sport accident. **a** K-wire osteosynthesis of 3rd, 4th and 5th metatarsal fractures. No visible callus formation at the 2-month postoperative follow-up. **b** Visible fracture gaps 4 months post-op. Still inadequate callus formation and persistent load-dependent pain. **c** No further callus formation nor proceeding consolidation 9 months post-op and persistent load-dependent pain. **d** Week 8 after PTH therapy. Consolidation of the fracture gaps. No pain under dynamic load

**Fig. 2:**
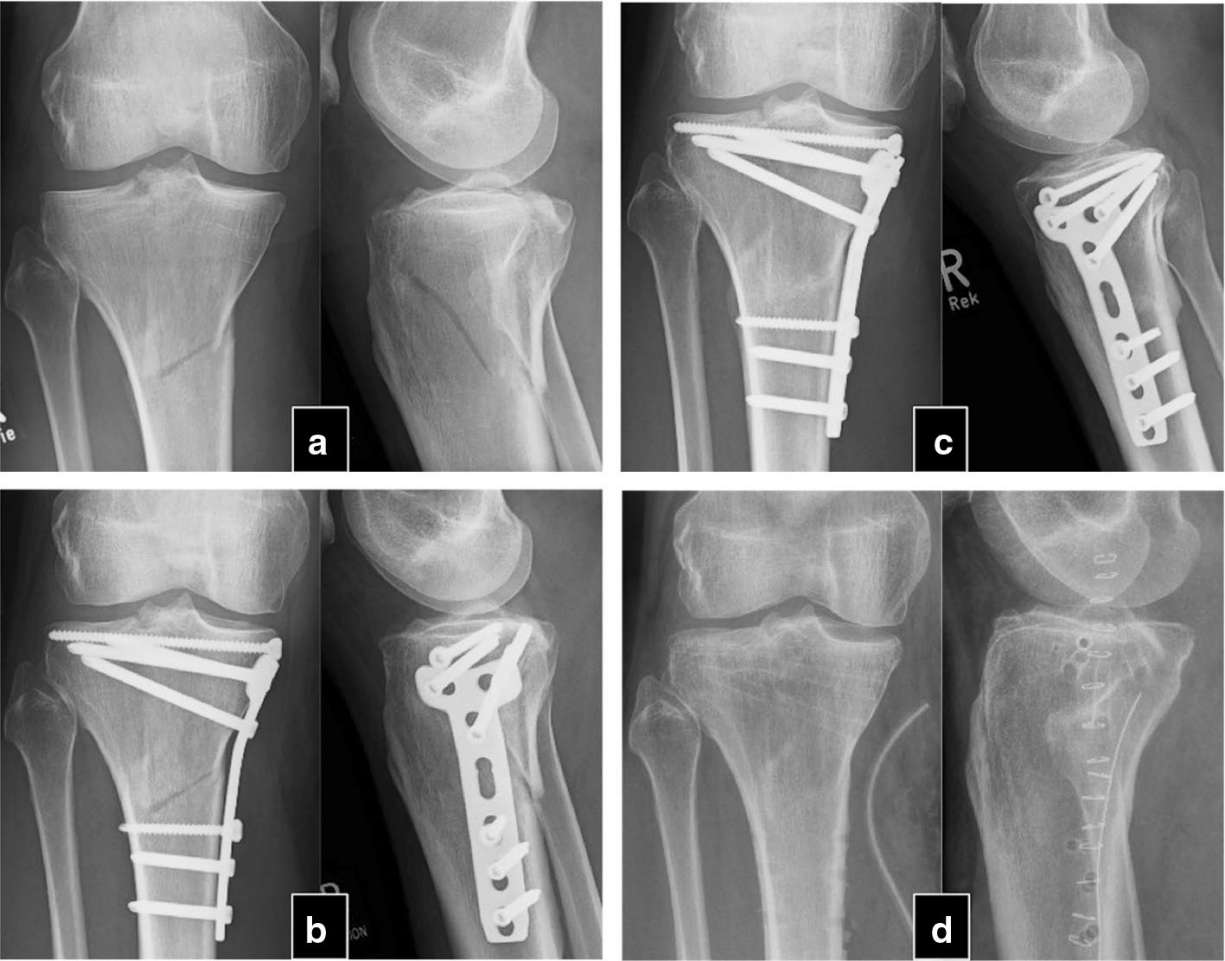
Thirty-nine-year-old male without other diseases or permanent medication with injury of the right leg in a motorcycle accident. **a** Fracture of the proximal tibia plateau and metaphysis. **b** Plate osteosynthesis 8 weeks postoperatively. No callus formation and bone bridging in the visible fracture gap. **c** 1 year post-op. Sclerosis of the fracture edges and still poor callus formation. Persisting load-dependent pain. **d** 6 months after initiation of PTH therapy and post-removal of implant material. Consolidation of fracture gaps and pain-free patient under dynamic load conditions

The mean time between the initial fracture and the PTH treatment was 24.3 ± 17.8 months (9–84). After an average of 4.1 ± 1.5 (2–6) months after PTH treatment, 30 of the 32 patients experienced a stable osseous consolidation of the nonunion and regained full, pain-free weight-bearing capacity of the fractured extremity. These 30 patients were treated between 4 and 10 (7.3 ± 1.5) weeks with PTH until first signs of healing appeared (Table 2). Usually, appearance of bone bridges and reduction in pain under dynamic load occurred simultaneously. Case examples are shown in Figs. 1 and 2.

**Table 2:** Results following PTH treatment

Time from fracture till PTH treatment (months)	9–84 (24.3 ± 17.8)
Treatment duration (weeks)	4–10 (7.3 ± 1.5)
Time to heal after PTH treatment (months) in 30 out of 32 patients	2–6 (4.1 ± 1.5)

Numbers represent min–max (mean ± SD)

In two patients, no signs of clinical and radiographical response were observed after 8 weeks of therapy with teriparatide, and therapy was stopped thereafter. The patients reported persisting pain in the nonunited extremities. In the latter in conventional radiography, no relevant signs of consolidation were observed within 6 months. The patients were an otherwise healthy 22-year-old woman with bilateral femur shaft fracture that had been operated twice (nail osteosynthesis and removal of material, cancellous bone grafting and reosteosynthesis) and an 80-year-old man with chronic heart disease, hypertension who suffered from a distal humerus fracture treated without surgical therapy.

In all patients, the therapy was well tolerated and no interruptions of medication were necessary as no side effects were experienced. Calcium and phosphate blood concentrations stayed within normal range, and there were no significant changes in serum creatinine during the whole observation period in all cases.

## Discussion

Teriparatide has been used as fracture prophylaxis in osteoporotic patients for many years. Few human studies and case reports exist that support the evidence found in animal studies that PTH has stimulating effects on fracture healing. Based on the idea that PTH (re)initiates the stage of remodelling by activating bone resorption and formation at the same time [2], we suppose that it could stimulate the consolidation of nonunions. To this moment the use of PTH as treatment for nonunions is off-label and has not been investigated in placebo-controlled studies, and it is questionable that it ever will be.

This study on 32 patients with nonunions at various locations after extremity fractures suggests that teriparatide can contribute to definitive healing of aseptic nonunions as 30 patients experienced consolidation of the previously fractured bones within a maximum of 6 months after PTH therapy. A failure of treatment occurred only in two patients, while the reasons for this remain concealed as both patients one young and otherwise healthy and one far aged morbid patient did not exhibit any possible predispositions to treatment failure. But, since many of the patients that experienced a healing of the corresponding nonunion suffered from one or more chronic disease, multimorbidity or higher age or gender does not appear to have a negative effect on the outcome of the teriparatide treatment.

The dosage of 20 µg per day was chosen based on the findings of a study showing that the accelerating effect in the healing of fractures in postmenopausal women was not dose dependent and occurred at a dose of 20 µg per day [6].

Since this study had no control group it cannot be proven that spontaneous consolidation would not have occurred without intervention. But the increasing number of recent reports about healing nonunions under treatment with teriparatide published by different groups concerning different fracture sites demands recognition [14–19]. Therefore, further studies with greater patient numbers should be conducted to evaluate the PTH effect on nonunions. The repetitive measurement of serum markers for bone healing as Xiaofeng et al. have shown in their recent case report of the treatment of nonunions of the tibia and the femur in one patient [14], could be one way to gain additional support for the positive effect of PTH on fracture healing.

Meanwhile, in otherwise hopeless situations of aseptic nonunions in patients with normal vitamin D levels, when the usual therapeutic options are exhausted, the treatment with PTH should be considered as a viable alternative.
